# Two Sides of the Same Coin? A Comparison between Internet-based and Paper-based Data Collection for Autism Quotient and Depression, Anxiety and Stress Scale

**DOI:** 10.1192/j.eurpsy.2024.1499

**Published:** 2024-08-27

**Authors:** D. Sönmez, Y. Abidi, T. R. Jordan

**Affiliations:** Psychology, Ibn Haldun University, Istanbul, Türkiye

## Abstract

**Introduction:**

The utilization of internet-based data collection in mental health research has gained popularity for its convenience and affordability. However, concerns often arise regarding the validity and reliability of data collected via the internet. The Autism Spectrum Quotient (AQ) is a self-report questionnaire to measure the traits associated with autism spectrum disorder (Baron-Cohen *et al*. J Autism Dev Disord, 2001; 31 5-17) and the online usage of AQ is common and conducted with large numbers of participants across many studies. However, the effect of using internet-based data collection for AQ rather than conventional paper-based procedures is unknown.

**Objectives:**

To address this issue, we conducted a study comparing the effectiveness of internet-based and paper-based data collection procedures for both the AQ and Depression Anxiety Stress Scale-21 (DASS-21, Lovibond & Lovibond, Behav Res Ther 1995; 33 335–343), which is also a prevalent mental health measurement in the literature and often used for online data collection (Zlomke, Comput Hum Behav 2009; 25 841-843). In addition, to compare internet-based and paper-based methods more fully, we included another variable (type of supervision) where a researcher was either present or absent during the completion of the questionnaires.

**Methods:**

A power analysis was conducted, and a minimum of 90 participants were needed to reach a medium effect size of .30 with an adequate power of .80 at a= .05. Accordingly, 96 participants were used and randomly assigned across 4 data collection groups: internet-based (supervision, no supervision) and paper-based (supervision, no supervision). In addition to a Demographic Form, AQ, and DASS-21 were used to obtain the data. Three independent variables were used in the current study: type of presentation (internet-based and paper-based) and type of supervision as between factors, and type of assessment as a within factor.

**Results:**

Using a 2 x 2 x 2 mixed design ANOVA, no significant main effects were found for any independent variables (all *p* > .33) or interaction (all *ps* > .17).

**Conclusions:**

The results of using AQ and DASS-21 were not altered by using internet-based or paper-based data collection procedures, suggesting that both methodologies are equally valid for this purpose. Moreover, these effects were also unaffected by the presence or absence of a researcher during data collection, suggesting that supervision by an authoritative figure does not alter the responses made.

**Disclosure of Interest:**

None Declared

